# *catena*-Poly[[dichloridomercury(II)]-*N*′-nicotinoylnicotinohydrazide]

**DOI:** 10.1107/S1600536812008884

**Published:** 2012-03-03

**Authors:** Teng Ma, Yuanlu Wang, Fengliang Wang, Fei Li

**Affiliations:** a College of Pharmacy, Nanjing Medical University, Nanjing 210029, People’s Republic of China

## Abstract

The title complex, [HgCl_2_(C_12_H_10_N_4_O_2_)]_*n*_, is composed of one Hg^II^ ion, one nnh ligand (nnh = *N*′-nicotinoylnicotinohydrazide) and two coordinated chloride ions. The Hg^II^ ion shows a distorted tetra­hedral geometry, being surrounded by two N atoms from two nnh ligands and two chloride ions. Due to the bridging role of nnh, the Hg^II^ atoms are connected into polymeric chains along the *c* axis, which are further inter­linked *via* N—H⋯O and C—H⋯Cl hydrogen-bonding inter­actions, forming a three-dimensional network.

## Related literature

For the coordination systems of *N*-donor heterocyclic groups, see: Zhang & Chen (2010 ▶); Ma *et al.* (2005 ▶); Tao *et al.* (2010 ▶).
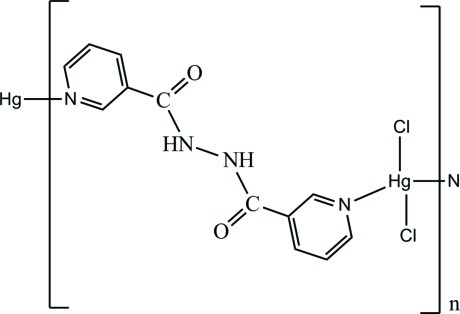


## Experimental

### 

#### Crystal data

[HgCl_2_(C_12_H_10_N_4_O_2_)]
*M* *_r_* = 513.73
Monoclinic, 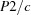

*a* = 7.2514 (4) Å
*b* = 4.7113 (3) Å
*c* = 21.8591 (11) Å
β = 103.394 (2)°
*V* = 726.47 (7) Å^3^
*Z* = 2
Mo *K*α radiation
μ = 10.97 mm^−1^
*T* = 296 K
0.30 × 0.26 × 0.22 mm

#### Data collection

Bruker SMART CCD area-detector diffractometer
Absorption correction: multi-scan (*SADABS*; Sheldrick, 1996 ▶) *T*_min_ = 0.137, *T*_max_ = 0.196
3510 measured reflections
1288 independent reflections
1244 reflections with *I* > 2σ(*I*)
*R*_int_ = 0.018

#### Refinement

*R*[*F* ^2^ > 2σ(*F* ^2^)] = 0.016
*wR*(*F* ^2^) = 0.038
*S* = 1.09
1288 reflections
96 parameters
H-atom parameters constrained
Δρ_max_ = 0.52 e Å^−3^
Δρ_min_ = −0.57 e Å^−3^

### 

Data collection: *SMART* (Bruker, 2007 ▶); cell refinement: *SAINT* (Bruker, 2007 ▶); data reduction: *SAINT*; program(s) used to solve structure: *SHELXS97* (Sheldrick, 2008 ▶); program(s) used to refine structure: *SHELXL97* (Sheldrick, 2008 ▶); molecular graphics: *DIAMOND* (Brandenburg, 1999 ▶); software used to prepare material for publication: *SHELXTL* (Sheldrick, 2008 ▶).

## Supplementary Material

Crystal structure: contains datablock(s) I, global. DOI: 10.1107/S1600536812008884/hp2029sup1.cif

Structure factors: contains datablock(s) I. DOI: 10.1107/S1600536812008884/hp2029Isup2.hkl

Additional supplementary materials:  crystallographic information; 3D view; checkCIF report

## Figures and Tables

**Table 1:** Hydrogen-bond geometry (Å, °)

*D*—H⋯*A*	*D*—H	H⋯*A*	*D*⋯*A*	*D*—H⋯*A*
C3—H3⋯Cl1^i^	0.93	2.81	3.558 (4)	138
N2—H2*A*⋯O1^ii^	0.86	2.15	2.844 (3)	137

Symmetry codes: (i) 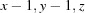; (ii) 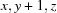.

**Fig. 1.**
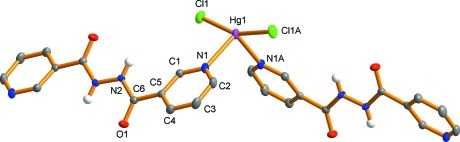
Coordination environment of HgII in the title complex showing displacement ellipsoids for all non-H atoms drawn at the 30% probability level. [Symmetry code (A): 1 - x, y, 3/2 - z.].

**Fig. 2.**
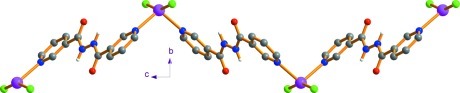
View of the one-dimensional chain.

**Fig. 3.**
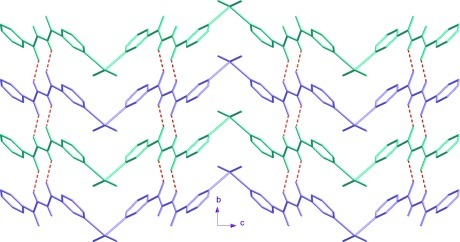
View of the two-dimensional layer via N–H···O hydrogen bonds (red dashed lines).

**Fig. 4.**
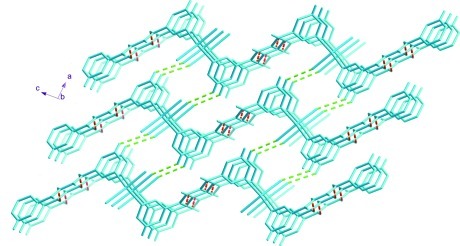
View of the three-dimensional network via C–H···Cl hydrogen bonds (green dashed lines).

## supplementary crystallographic information

### Comment

Flexible ligands containing *N*-donor heterocyclic groups, such as pyridyl, pyrazinyl, and triazolyl (see: Zhang *et al.*, 2010; Ma *et al.*, 2005; Tao *et al.*, 2010), have been widely studied in the realm of metal-organic coordination assemblies. With regard to this, *N*'-nicotinoylnicotinohydrazide (nnh), an interesting ligand with flexible spacer and multiple binding sites, has attract our attention. Herein, we report the title complex [Hg(nnh)Cl_2_]*_n_*, which crystallizes in the monoclinic space group *P*2/*c*, and shows a one-dimensional polymeric array and H-bonding supramolecular network.

As shown in Fig.1, the asymmetric unit of the complex is provided by a Hg^II^ center, one nnh ligand and two chloride ions. The Hg^II^ ion is tetra-coordinated to two nitrogen atoms from two nnh ligands with the Hg—N distance of 2.475 (2) Å, as well as two chloride ions with the Hg—Cl distance of 2.3405 (9) Å. The adjacent Hg centers are bridged by the nnh ligands to afford a one-dimensional zigzag chain with the Hg···Hg separation of *ca* 12.8371 (6) Å (see Fig. 2).

Notably, H-bonding interactions do play a decisive role in the crystal packing arrangement. As shown in Fig. 3, the adjacent one-dimensional arrays are linked to form a two-dimensional layer *via* N2—H2A···O2^i^ [symmetry operation (i) = *x*, 1 + *y*, *z*] hydrogen bonding between the nnh ligands from different chains. Furthermore, such two-dimensional layers are interlinked by the weak hydrogen bonds C3—H3···Cl^ii^ [symmetry operation (ii) = -1 + *x*, -1 + *y*, *z*] to generate a three-dimensional supramolecular network (see Fig. 4).

### Experimental

A CH_3_OH solution (10 ml) of nnh (24.2 mg, 0.1 mmol) was carefully layered onto an aqueous solution of HgCl_2_ (27.1 mg, 0.1 mmol) in a straight glass tube. After evaporating the solvents slowly for *ca* one month, suitable yellow block single crystals for X-ray analysis were produced.

### Refinement

All H atoms were initially located in a difference Fourier map, which were then constrained to an ideal geometry, and refined as riding atoms: C—H = 0.93 (CH_aromatic_) and N—H = 0.86, with *U*iso(H) = 1.2*U*eq (C) and *U*iso(H) = 1.5*U*eq (N).

### Crystal data

**Table:**

[HgCl_2_(C_12_H_10_N_4_O_2_)]	*F*(000) = 480
*M**_r_* = 513.73	*D*_x_ = 2.349 Mg m^−^^3^
Monoclinic, *P*2/*c*	Mo *K*α radiation, λ = 0.71073 Å
Hall symbol: -P 2yc	Cell parameters from 2648 reflections
*a* = 7.2514 (4) Å	θ = 2.9–27.9°
*b* = 4.7113 (3) Å	µ = 10.97 mm^−^^1^
*c* = 21.8591 (11) Å	*T* = 296 K
β = 103.394 (2)°	BLOCK, yellow
*V* = 726.47 (7) Å^3^	0.30 × 0.26 × 0.22 mm
*Z* = 2	

### Data collection

**Table:**

Bruker SMART CCD area-detector diffractometer	1288 independent reflections
Radiation source: fine-focus sealed tube	1244 reflections with *I* > 2σ(*I*)
Graphite monochromator	*R*_int_ = 0.018
phi and ω scans	θ_max_ = 25.0°, θ_min_ = 1.9°
Absorption correction: multi-scan (*SADABS*; Sheldrick, 1996)	*h* = −8→8
*T*_min_ = 0.137, *T*_max_ = 0.196	*k* = −5→5
3510 measured reflections	*l* = −14→26

### Refinement

**Table:**

Refinement on *F*^2^	Primary atom site location: structure-invariant direct methods
Least-squares matrix: full	Secondary atom site location: difference Fourier map
*R*[*F*^2^ > 2σ(*F*^2^)] = 0.016	Hydrogen site location: inferred from neighbouring sites
*wR*(*F*^2^) = 0.038	H-atom parameters constrained
*S* = 1.09	*w* = 1/[σ^2^(*F*_o_^2^) + (0.0224*P*)^2^ + 0.0147*P*] where *P* = (*F*_o_^2^ + 2*F*_c_^2^)/3
1288 reflections	(Δ/σ)_max_ = 0.001
96 parameters	Δρ_max_ = 0.52 e Å^−^^3^
0 restraints	Δρ_min_ = −0.57 e Å^−^^3^

### Special details

**Table:**

Geometry. All e.s.d.'s (except the e.s.d. in the dihedral angle between two l.s. planes) are estimated using the full covariance matrix. The cell e.s.d.'s are taken into account individually in the estimation of e.s.d.'s in distances, angles and torsion angles; correlations between e.s.d.'s in cell parameters are only used when they are defined by crystal symmetry. An approximate (isotropic) treatment of cell e.s.d.'s is used for estimating e.s.d.'s involving l.s. planes.
Refinement. Refinement of *F*^2^ against ALL reflections. The weighted *R*-factor *wR* and goodness of fit *S* are based on *F*^2^, conventional *R*-factors *R* are based on *F*, with *F* set to zero for negative *F*^2^. The threshold expression of *F*^2^ > σ(*F*^2^) is used only for calculating *R*-factors(gt) *etc*. and is not relevant to the choice of reflections for refinement. *R*-factors based on *F*^2^ are statistically about twice as large as those based on *F*, and *R*- factors based on ALL data will be even larger.

### Fractional atomic coordinates and isotropic or equivalent isotropic displacement parameters (Å^2^)

**Table:**

	*x*	*y*	*z*	*U*_iso_*/*U*_eq_	
Hg1	0.5000	1.07616 (3)	0.7500	0.03638 (8)	
Cl1	0.81805 (12)	1.1749 (2)	0.79628 (5)	0.0524 (2)	
O1	0.7884 (3)	0.1092 (4)	0.98476 (12)	0.0385 (5)	
N1	0.4508 (3)	0.7488 (5)	0.83334 (12)	0.0348 (6)	
N2	0.9166 (4)	0.5412 (5)	0.97954 (13)	0.0293 (6)	
H2A	0.9048	0.7096	0.9639	0.035*	
C1	0.6042 (4)	0.6598 (6)	0.87486 (15)	0.0305 (6)	
H1	0.7211	0.7361	0.8730	0.037*	
C2	0.2825 (5)	0.6416 (8)	0.83653 (16)	0.0405 (8)	
H2	0.1741	0.7054	0.8082	0.049*	
C3	0.2644 (5)	0.4401 (7)	0.88032 (18)	0.0423 (8)	
H3	0.1454	0.3698	0.8815	0.051*	
C4	0.4239 (4)	0.3429 (7)	0.92245 (15)	0.0345 (7)	
H4	0.4146	0.2023	0.9514	0.041*	
C5	0.5987 (4)	0.4593 (6)	0.92072 (15)	0.0267 (6)	
C6	0.7733 (4)	0.3541 (6)	0.96446 (14)	0.0264 (6)	

### Atomic displacement parameters (Å^2^)

**Table:**

	*U*^11^	*U*^22^	*U*^33^	*U*^12^	*U*^13^	*U*^23^
Hg1	0.03059 (11)	0.04571 (12)	0.02898 (11)	0.000	−0.00099 (8)	0.000
Cl1	0.0347 (4)	0.0712 (6)	0.0475 (5)	−0.0132 (4)	0.0020 (4)	−0.0076 (5)
O1	0.0319 (12)	0.0234 (10)	0.0553 (15)	0.0000 (8)	0.0000 (11)	0.0057 (9)
N1	0.0289 (13)	0.0411 (14)	0.0303 (14)	0.0040 (11)	−0.0016 (11)	−0.0001 (12)
N2	0.0235 (12)	0.0220 (11)	0.0361 (15)	−0.0008 (9)	−0.0057 (11)	0.0066 (10)
C1	0.0271 (15)	0.0304 (14)	0.0316 (16)	−0.0018 (12)	0.0017 (13)	−0.0014 (13)
C2	0.0293 (17)	0.0529 (18)	0.0331 (18)	0.0022 (14)	−0.0053 (15)	−0.0012 (15)
C3	0.0234 (17)	0.058 (2)	0.042 (2)	−0.0087 (14)	0.0019 (15)	−0.0062 (16)
C4	0.0292 (17)	0.0387 (15)	0.0339 (17)	−0.0052 (13)	0.0041 (14)	−0.0009 (14)
C5	0.0254 (15)	0.0255 (13)	0.0279 (16)	−0.0010 (11)	0.0032 (13)	−0.0054 (12)
C6	0.0251 (15)	0.0239 (13)	0.0295 (16)	0.0008 (11)	0.0051 (13)	−0.0018 (12)

### Geometric parameters (Å, º)

**Table:**

Hg1—Cl1	2.3405 (9)	C1—C5	1.385 (5)
Hg1—Cl1^i^	2.3405 (9)	C1—H1	0.9300
Hg1—N1^i^	2.475 (2)	C2—C3	1.376 (5)
Hg1—N1	2.475 (2)	C2—H2	0.9300
O1—C6	1.232 (3)	C3—C4	1.379 (5)
N1—C1	1.330 (4)	C3—H3	0.9300
N1—C2	1.338 (4)	C4—C5	1.390 (4)
N2—C6	1.344 (4)	C4—H4	0.9300
N2—N2^ii^	1.383 (5)	C5—C6	1.484 (4)
N2—H2A	0.8601		
			
Cl1—Hg1—Cl1^i^	157.08 (6)	N1—C2—C3	122.1 (3)
Cl1—Hg1—N1^i^	98.41 (6)	N1—C2—H2	118.9
Cl1^i^—Hg1—N1^i^	95.81 (6)	C3—C2—H2	118.9
Cl1—Hg1—N1	95.81 (6)	C2—C3—C4	119.6 (3)
Cl1^i^—Hg1—N1	98.41 (6)	C2—C3—H3	120.2
N1^i^—Hg1—N1	102.92 (11)	C4—C3—H3	120.2
C1—N1—C2	118.3 (3)	C3—C4—C5	118.7 (3)
C1—N1—Hg1	117.28 (19)	C3—C4—H4	120.7
C2—N1—Hg1	124.2 (2)	C5—C4—H4	120.7
C6—N2—N2^ii^	119.0 (3)	C1—C5—C4	117.9 (3)
C6—N2—H2A	120.5	C1—C5—C6	122.1 (3)
N2^ii^—N2—H2A	120.5	C4—C5—C6	119.9 (3)
N1—C1—C5	123.3 (3)	O1—C6—N2	121.8 (3)
N1—C1—H1	118.3	O1—C6—C5	122.4 (3)
C5—C1—H1	118.3	N2—C6—C5	115.8 (2)
			
Cl1—Hg1—N1—C1	−12.5 (2)	C2—C3—C4—C5	2.1 (5)
Cl1^i^—Hg1—N1—C1	−174.5 (2)	N1—C1—C5—C4	1.1 (5)
N1^i^—Hg1—N1—C1	87.6 (2)	N1—C1—C5—C6	177.0 (3)
Cl1—Hg1—N1—C2	172.4 (2)	C3—C4—C5—C1	−2.5 (5)
Cl1^i^—Hg1—N1—C2	10.4 (3)	C3—C4—C5—C6	−178.5 (3)
N1^i^—Hg1—N1—C2	−87.6 (3)	N2^ii^—N2—C6—O1	−2.0 (5)
C2—N1—C1—C5	0.8 (5)	N2^ii^—N2—C6—C5	179.5 (3)
Hg1—N1—C1—C5	−174.6 (2)	C1—C5—C6—O1	−147.0 (3)
C1—N1—C2—C3	−1.3 (5)	C4—C5—C6—O1	28.8 (4)
Hg1—N1—C2—C3	173.8 (3)	C1—C5—C6—N2	31.5 (4)
N1—C2—C3—C4	−0.2 (5)	C4—C5—C6—N2	−152.7 (3)

Symmetry codes: (i) −*x*+1, *y*, −*z*+3/2; (ii) −*x*+2, −*y*+1, −*z*+2.

### Hydrogen-bond geometry (Å, º)

**Table:**

*D*—H···*A*	*D*—H	H···*A*	*D*···*A*	*D*—H···*A*
C3—H3···Cl1^iii^	0.93	2.81	3.558 (4)	138
N2—H2*A*···O1^iv^	0.86	2.15	2.844 (3)	137

Symmetry codes: (iii) *x*−1, *y*−1, *z*; (iv) *x*, *y*+1, *z*.

## References

[bb1] Brandenburg, K. (1999). *DIAMOND* Crystal Impact GbR, Bonn, Germany.

[bb2] Bruker (2007). *SMART* and *SAINT* Bruker AXS Inc., Madison, Wisconsin, USA.

[bb3] Ma, B.-Q., Mulfort, K. L. & Hupp, J. T. (2005). *Inorg. Chem.* **44**, 4912–4914. 10.1021/ic050452i 15998017

[bb4] Sheldrick, G. M. (1996). *SADABS* University of Göttingen, Germany.

[bb5] Sheldrick, G. M. (2008). *Acta Cryst.* A**64**, 112–122. 10.1107/S0108767307043930 18156677

[bb6] Tao, Y., Li, J.-R., Chang, Z. & Bu, X.-H. (2010). *Cryst. Growth Des.* **10**, 564–574.

[bb7] Zhang, S.-S. & Chen, L.-J. (2010). *Acta Cryst.* E**66**, m1456. 10.1107/S1600536810042406 PMC3009079 21588874

